# Strategies to stabilize compact folding and minimize aggregation of antibody-based fragments

**DOI:** 10.4236/abb.2013.44A011

**Published:** 2013-04

**Authors:** Diana Gil, Adam G. Schrum

**Affiliations:** Department of Immunology, Mayo Clinic College of Medicine, Rochester, USA

**Keywords:** Antibody, Immunoglobulin, Fragment, Fab, Therapy, Aggregation, Conformation, Protein Folding, Osmolyte

## Abstract

Monoclonal antibodies (mAbs) have proven to be useful for development of new therapeutic drugs and diagnostic techniques. To overcome the difficulties posed by their complex structure and folding, reduce undesired immunogenicity, and improve pharmacokinetic properties, a plethora of different Ab fragments have been developed. These include recombinant Fab and Fv segments that can display improved properties over those of the original mAbs upon which they are based. Antibody (Ab) fragments such as Fabs, scFvs, diabodies, and nanobodies, all contain the variable Ig domains responsible for binding to specific antigenic epitopes, allowing for specific targeting of pathological cells and/or molecules. These fragments can be easier to produce, purify and refold than a full Ab, and due to their smaller size they can be well absorbed and distributed into target tissues. However, the physicochemical and structural properties of the immunoglobulin (Ig) domain, upon which the folding and conformation of all these Ab fragments is based, can limit the stability of Ab-based drugs. The Ig domain is fairly sensitive to unfolding and aggregation when produced out of the structural context of an intact Ab molecule. When unfolded, Ab fragments may lose their specificity as well as establish non-native interactions leading to protein aggregation. Aggregated antibody fragments display altered pharmacokinetic and immunogenic properties that can augment their toxicity. Therefore, much effort has been placed in understanding the factors impacting the stability of Ig folding at two different levels: 1) intrinsically, by studying the effects of the amino acid sequence on Ig folding; 2) extrinsically, by determining the environmental conditions that may influence the stability of Ig folding. In this review we will describe the structure of the Ig domain, and the factors that impact its stability, to set the context for the different approaches currently used to achieve stable recombinant Ig domains when pursuing the development of Ab fragment-based biotechnologies.

## 1. INTRODUCTION

Abs are secreted glycoproteins of ~150 kDa that represent the soluble form of the antigen receptor of B cells [1]. Their function is to bind to specific antigens (Ag) from pathogens, mediating Ag neutralization and clearance in cooperation with other components of the immune system [2]. The majority of Abs are covalent tetramers composed of two heavy (H) and two (L) light chains. At the N-terminus of each H/L heterodimer, the Ag binding site results from the combination of two different sets of three complementary determining regions (CDRs) belonging to the H and L chains. The combinatorial diversity provided by the hyper-variable sequences in the CDR determines the unique specificity of each Ab for its Ag [3]. Since the development of hybridoma technology to produce mAbs in the 1970’s [4], much attention has focused on exploiting the potential of mAbs as biopharmaceutical drugs and diagnostic tools [5]. Exquisite specificity and strong affinity for their Ags are the key features that make mAbs so attractive for the pharmaceutical industry. Binding of Abs to specific proteins at the cell surface allows the targeting of pathologic tissues like tumoral masses. Bound Abs can directly reduce cell viability by blocking surface receptors key for cell survival, by activation of complement-mediated cytotoxicity, and/or by signaling through their target receptors and inducing apoptosis. On the other hand, Ab specificity can be exploited to achieve targeted delivery of coupled enzymes, toxins, viruses, radioisotopes, among many other payloads, in order to achieve either therapy or imaging in the tissue of choice. Moreover Abs can be used to specifically bind and neutralize toxins and poisons.

A key limiting factor in the application of mAbs as bio-drugs is their pharmacokinetic properties [6]. A fine balance between adequate tissue penetration/retention and paced excretion/degradation is required to optimize the therapeutic effects of mAbs. Abs are large molecules that circulate in serum with long half-lives thanks to the interaction of their Fc portion with serum proteins like the neonatal Fc receptor (FcRn) [7]. However, long half-life could become pathogenic when using mAbs coupled to isotopes or toxins, due to a low rate of systemic clearance. On the other hand, the size of mAbs (~150 kDa) hinders their capacity to penetrate tissues, reducing the efficacy of Ab-therapies against solid tumors. Since some other undesired effects of mAbs are related with the Fc portion (*i.e*., “cytokine storm”, caused by massive activation of lymphocytes by Fc/FcR crosslinking) [8,9], one solution to the limitations imposed by the size and the half-life of mAbs has been to develop Ab fragments lacking the Fc portion. Fc-deficient Ab fragments display improved tissue penetration, revealing an inverse correlation between size and capacity to reach the targeted tissue [10]. On the other hand, retention time in liver and other tissues, together with serum half-life, are reduced by the lack of Fc, since Fc-deficient Ab fragments fail to interact with Fc receptors present at cell surfaces or soluble in the plasma. A faster turnover for Ab fragments translates into reduced toxicity due to payloads like radioisotopes when compared with intact Abs [11]. This property is especially desirable for clinical imaging, since faster clearance of Ab fragments not only reduces toxicity but also background activity of the radioactive probes [12]. However, reducing the Ab fragment to a size in the range of 30 kDa can over-accelerate its clearance from tissues, and may compromise its potential therapeutic effects [13].

There has been great technological advancement in the field of development of new Ab-fragment based technologies, including generation of recombinant Fab fragments and nanobodies, the latter composed of a single functional variable domain originating from a parent mAb [13]. While the size and valency of Ab fragments is tailored to suit best the application of choice (tissue imaging, tumor targeting, etc.), in every case the Ig domain(s) that structure and provide functionality to these molecules may unfold during production, storage and/or administration. Depending on the solubility of specific Ig domains and the kinetics of refolding, Ig unfolding can be either a reversible or irreversible process [14]. Ig unfolding could eventually lead to either complete denaturation followed by precipitation, or to aggregation of soluble Ab fragments. In any case, when Ig folding becomes compromised, the therapeutic and pharmacokinetic properties of the Ab fragment become altered, and could even result in toxicity when administered to patients. Only empirical studies monitoring unfolding and aggregation of each Ab-based fragment ensures the establishment of the right conditions required to minimize the amount of unfolded/aggregated Ig molecules [15]. However, the multitude of studies describing the stability of different Ab fragments, and their response to changes in their environment, allows the establishment of common strategies at three levels: 1) Engineering of covalent bonds to stabilize the interaction of H and L chains in the Ab fragment; 2) sequence mutagenesis to increase stability and solubility of the Ig domains composing the Ab fragment; 3) buffering the environmental conditions that cause Ig instability during production, storage and utilization of Ab fragments.

## 2. INTACT Ab STRUCTURE AND THE Ig FOLD

**Figure 1:**
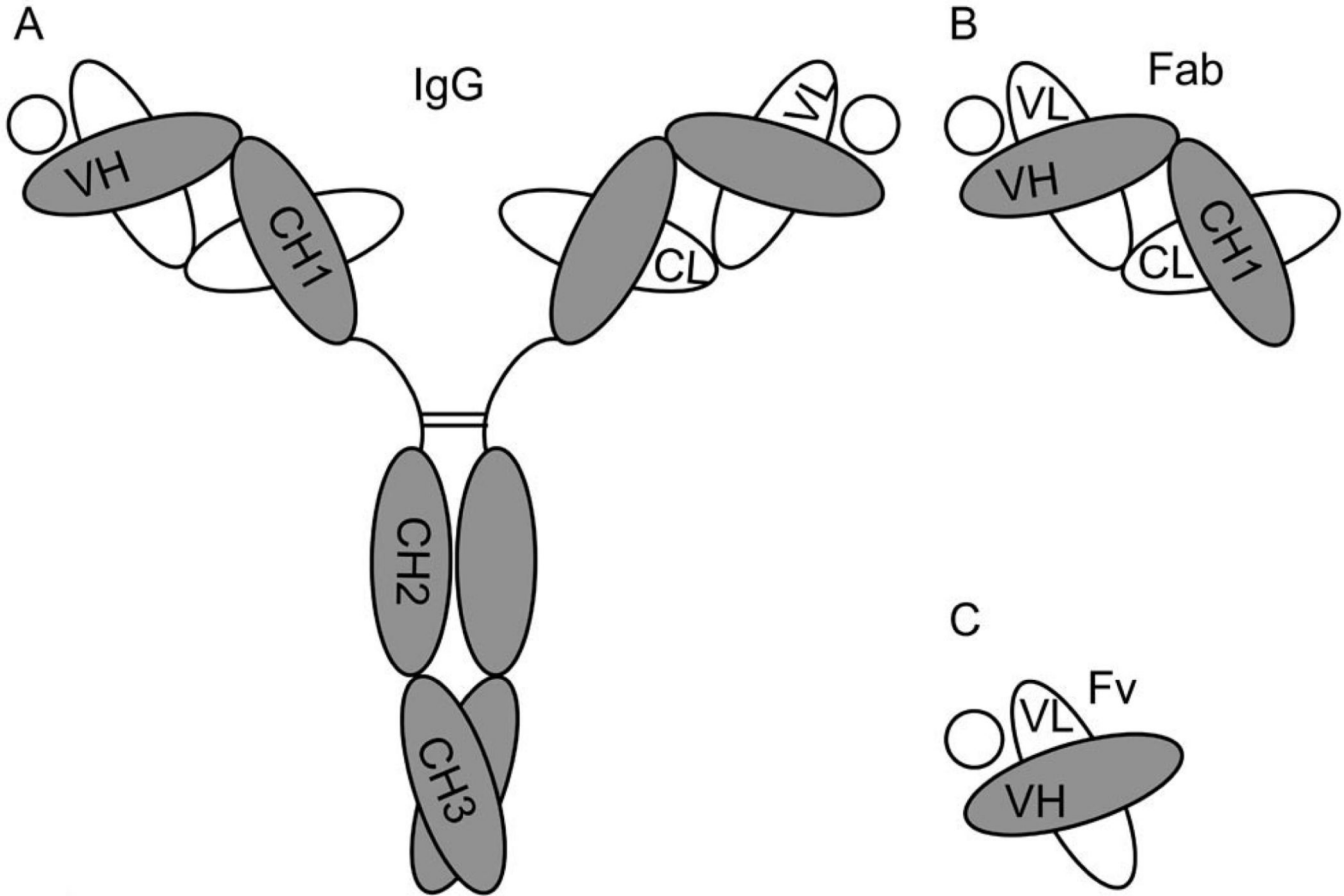
Diagrams depicting the structure of (A) the intact Ab molecule (IgG); (B) a Fab fragment; and (C) a Fv fragment. The circles in each panel represent the specific Ag of the depicted Ab molecule.

The Ab molecule is a tetramer composed of two identical copies of a glycosylated heterodimer that contains a heavy (H) and a light (L) chain covalently linked via disulfide bond. The H-L heterodimers associate in turn through their H chains by additional disulfide bonds (Figure 1A). The Ig domain is the unit of tertiary structure found repeatedly along the amino acid sequences of the H and L chains [2,16–18] (Figure 1A). The Ig domain includes from 110 to 130 residues that fold into a characteristic tertiary structure called the β-barrel. The β-barrel is composed of two β-sheets that are held together by an intra-chain disulfide bond. Each β-sheet is composed of 3 – 4 anti-parallel β-strands of 5 – 10 amino acids each. The β-strands are connected by stretches of amino acids that do not follow such secondary structure and form connecting loops of variable length. Along the sequence of the β-sheet, hydrophobic and hydrophilic residues alternate pointing their side chains in opposite directions. This way, when the two β-sheets are paired by the disulfide bond, a hydrophobic core and a hydrophilic surface are established for the β-barrel. In this structure the connecting loops define the extremes of the barrel. In the variable (V) Ig domains of the H and L chain, the N terminal loops include three hyper-variable sequences or complementary determining regions (CDR) that form the epitope binding site. When comparing the sequence homology among different Ig domains, hydrophobic residues are found in conserved positions along the β-strands.

The Ig domain is also found in many other proteins besides Abs. A protein domain is classified as Ig or Ig-related when its structure, size and amino acid sequence are similar to the β-barrel found in Abs. All the Ig containing proteins constitute an evolutionary related group of proteins denominated the Ig-superfamily. Ig-superfamily members include surface proteins in cells of the immune and nervous system that regulate processes like cellular recognition and tissue adhesion. Interestingly, there are no residues conserved with 100% identity within the Ig domain in the Ig superfamily, not even the cysteines involved in the intra-chain disulfide bond, which are substituted by hydrophobic residues in some Ig-related proteins like CD2, CD4 and LFA3 [18]. Thus, the hydrophobic core of the β-barrel sustains the Ig folding, and the rest of the structure allows for sequence variability that in turn permits a multiplicity of functions along the Ig-superfamily members. Still, there is a common functional feature in the Ig-superfamily, since most of the members function as receptors or adhesion molecules and utilize the Ig domains for recognition of unique structures.

The quaternary structure of the Ab molecule is stabilized by the disulfide bonds found along the H and L chains (Figure 1A). Additionally, a series of hydrophobic interactions between inter-chain Ig domains also contribute to the stability of the whole molecule. These interactions are based on complementary surfaces in the involved β-barrels that contain some hydrophobic residues [14,19]. By juxtaposing these hydrophobic complementary surfaces, the Ig domains stabilize their folding by hiding non-polar regions from the aqueous environment [20]. Following the IgG molecule as an Ab prototype, the VH and VL, the CH1 and the CL, and the CH3 Ig domains interact through hydrophobic patches at their interface (Figure 1A) [21,22]. One study showed that a complementary pair of CH1/CL domains was as stable as the whole intact Fab fragment (VH-CH1/VL-CL) in the presence of a denaturing agent, suggesting that C-domains can make the most important contributions for the stability of Ig folding in Fab fragments [19]. Although this enhanced contribution of CH1/CL pairing is not completely understood, it has been suggested that the Ig domains involved present bigger complementary surfaces with more hydrophobic residues that interact with a different angle than in the interface of VH/VL complementary pairs [14,19]. Finally, in the IgG molecule, the CH2 domains that do not interact directly through complementary surfaces, establish weak carbohydrate interactions at the level of the oligosaccharides present in these domains that can also contribute to structural stability of the Ab molecule [23].

## 3. UNDERSTANDING Ig UNFOLDING IN Ab FRAGMENTS

**Figure 2:**
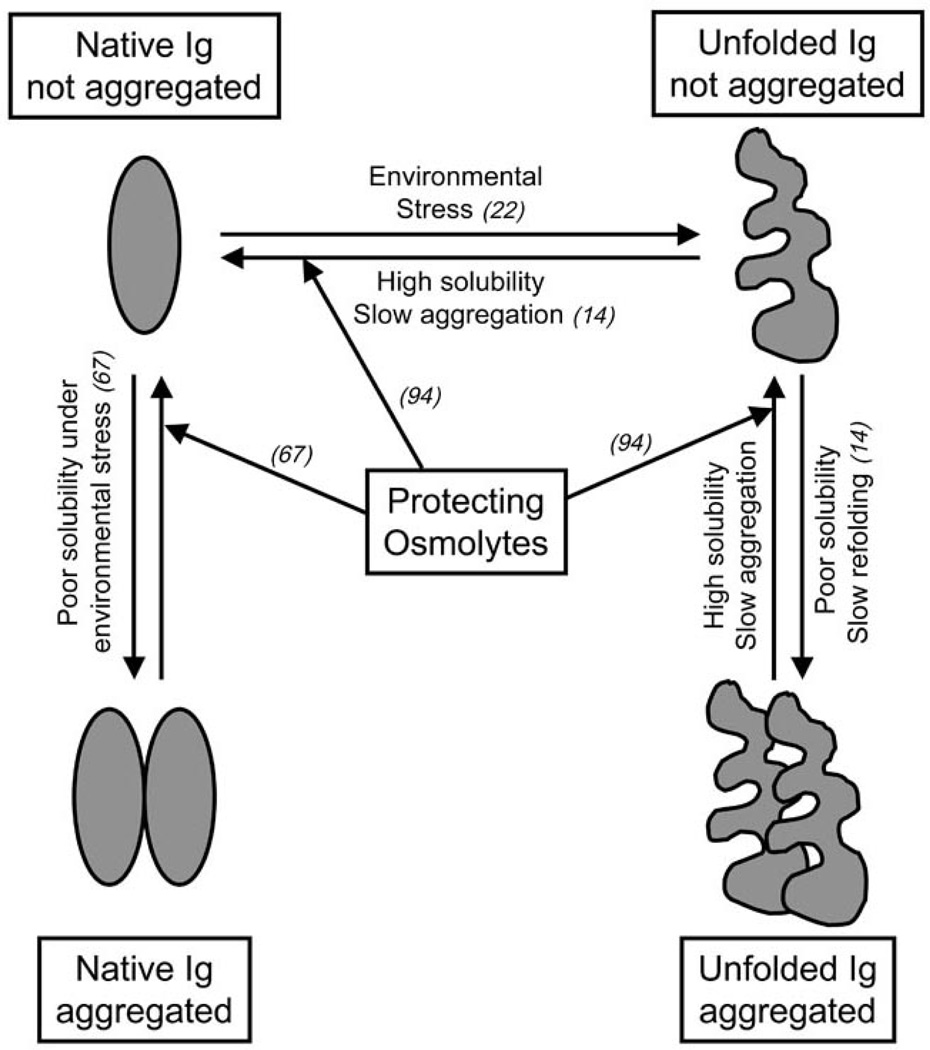
Ig unfolding and aggregation pathways. The scheme summarizes the pathways discussed along the text. Reference numbers of relevant articles describing these pathways are included in brackets.

Proteins in solution, and by extension the Ig domains in Abs and Ab fragments, are in equilibrium between their folded (native) and unfolded states (Figure 2). While the transition between these two states does not involve covalent interactions, all other non-covalent interactions that fold the Ab are at play, such as hydrogen bonds, electrostatic interactions, hydrophobic repulsion, and van der Waals forces [24]. Conditions like temperature, protein concentration, ionic strength, and pH impact the physico-chemical properties of the polypeptides, other solutes, and water of the solution, modulating non-covalent interactions and the folding equilibrium of proteins [22]. Ab-based proteins tend to unfold when exposed to environmental stress like high concentration, extreme pH, changes in ionic strength and temperature, lyophilization and/or re-hydration, freezing/thawing cycles, agitation, and prolonged storage (Figure 2). During the production, distribution and/or utilization of Abs and Ab fragments as therapeutic drugs or diagnostic tools, fluctuations in conditions like those mentioned above may occur. Once unfolded, the Ig domains may drive the aggregation of the Ab fragment as a mechanism to minimize entropy and free energy (Figure 2). Aggregated Ab fragments display increased immunogenicity and toxicity, while therapeutic and pharmacokinetic properties are perturbed. Thus, understanding the molecular mechanisms behind Ig unfolding and aggregation becomes key in the context of the development of biotechnology based on Ab fragments. Ig domains display more stable folding when present in intact Abs than as Ab fragments or individual domains [19,25]. This higher Ig stability is proposed to result from an additive effect of the combination of the multiple interactions established along the quarternary structure of the Ab: non-covalent interactions between complementary Ig domains, sugar interaction in glycosylated domains, and the disulfide bonds that covalently link H and L chains [26]. All these interactions determine that the kinetics of unfolding of an intact Ab molecule are relatively slow, but unfolded/aggregated moieties can accumulate over time [14]. Ig domains remain relative stable when in Fab fragments (Figure 1B), since some of the stabilizing interactions of the intact Ab are still present. Not surprisingly, the V Ig domains that compose monomeric Fv fragments (Figure 1C) are less stable than when included in Fab fragments [27], with elevated tendencies to unfold and aggregate. The cause of this instability seems to be related to the absence of additional complementary between domains, and disulfide bonds that in Fabs are supplied by the additional CH/CL pairs.

## 4. STRATEGIES TO INCREASE STABILITY OF Ig FOLDING IN Ab FRAGMENTS BASED ON COVALENT BONDS: ENGINEERING OF DISULFIDE BONDS AND PEPTIDE LINKERS

**Figure 3:**
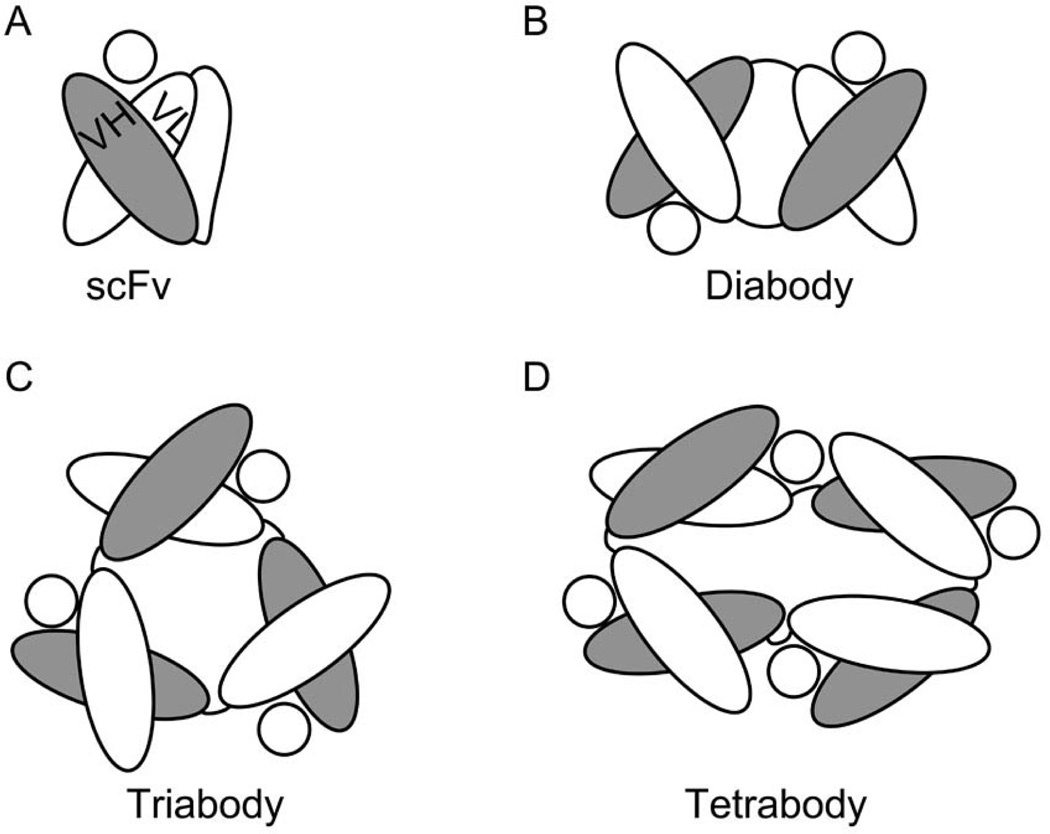
Diagrams depicting the structure of (A) an scFv fragment (IgG); and the derived (B) diabody; (C) triabody; and (D) tetrabody. The circles in each panel represent the specific Ag of the depicted Fv.

Given the interest in developing Ab fragments smaller than a Fab, stabilization of Ig folding in Fv fragments has been pursued by different approaches. The design of a covalent disulfide bond between VH and VL domains of the Fv (dsFv) was one of the first strategies employed to reinforce the V interface [28,29]. Another way to stabilize Fv fragments is to link the VH and VL chains with a flexible peptide sequence resistant to endopeptidases, generating single chain Fv (scFv) fragments (Figure 3A) [30,31]. Additionally sc-dsFv fragments have been developed by combining both disulfide bonding and peptide linking of VH and VL domains [32]. From these fragments, the most employed scFv still displays a tendency to unfold at the V interface, resulting in sub-optimal stability of the Ig domains [33]. When this happens, a phenomenon known as “protein domain swapping” can occur, wherein complementary Ig domains from adjacent scFv molecules interact with each other to result in scFv oligomerization [26]. This protein domain swapping has been described in different types of proteins other than Igs [34–36]. Indeed, Ig domain swapping can be engineered and optimized as a mechanism for controlling the precise oligomerization of scFv molecules [37,38]. Depending on the length of the peptide linker and the amino acid sequence of the Ab, short linkers that impede the proper rotation of the complementary Ig domains covalently linked to establish an interface, promote the swap of the same Ig domain between two, three or even four molecules of scFv (Figure 3A). The resulting diabodies, triabodies and tetrabodies (Figures 3B and D) display stable Ig folding and functional Ag recognition. On the other hand, they present different pharmacokinetic properties than the parental scFvs, such as stronger multivalent binding to the targeted epitope and prolonged retention in tissue [13,39]. An interesting application of the Ig domain swapping in scFv molecules is the development of bispecific diabodies [26]. A dimer of Fabs of different specificities can be formed when the VHA is linked to VLB in the scFv (A and B referring to different epitope specificities recognized by different FvA and FvB fragments) [40]. Bispecific diabodies are designed to either recognize epitopes located in the same antigenic structure, to increase binding to the antigen, or to recognize epitopes belonging to different Ags, in which case the diabody will crosslink/juxtapose otherwise separate Ags. The crosslinking properties of bispecific diabodies have been specially applied in the field of cancer therapeutics [13,41]. In some cases the diabodies are designed to promote the contact between tumor cells and cytotoxic effector cells of the immune system, such as cytotoxic T lymphocytes (CTLs) or Natural Killer (NK) cells, In other strategies, diabodies have been utilized to deliver specific toxins, radioactive haptens, or adenoviral gene delivery vehicles as a payload to tumor cells, resulting in their destruction.

## 5. ENGINEERING Ig FOLDING STABILITY IN MONOMERIC Ab FRAGMENTS: LESSONS FROM HEAVY CHAIN Abs FOUND IN CAMELIDS AND SHARKS

In spite of the advantages of the multimeric Ab fragments described above, there is a strong interest in the field of Ab-based therapeutics to develop small monomeric Ab fragments consisting of a single V domain [42–45]. The special attraction of so-called nanobodies is their minimal size that turns them into the simplest building block to link Ag specificity to a multiplicity of partners like enzymes, toxins, isotopes, viral particles, liposomes, ligands, receptors, and probes [44]. Taking into consideration the hydrophobic complementary surfaces of VH and VL Ig domains described above, one challenge to develop nanobody technology has been to obtain stable β-barrel structures for the single V domains in the absence of the complementary Ig domain. The additional challenge is to generate nanobodies with high affinity for their Ags. Although epitope recognition can be retained in a few lone VH domains in absence of their cognate VL, in these cases the affinity drops one to three orders of magnitude [46,47]. Additionally, these single VH domains display a high tendency to unfold, aggregate and precipitate out of solution, hampering the initial enthusiasm on the development of nanobodies [13,44].

**Figure 4:**
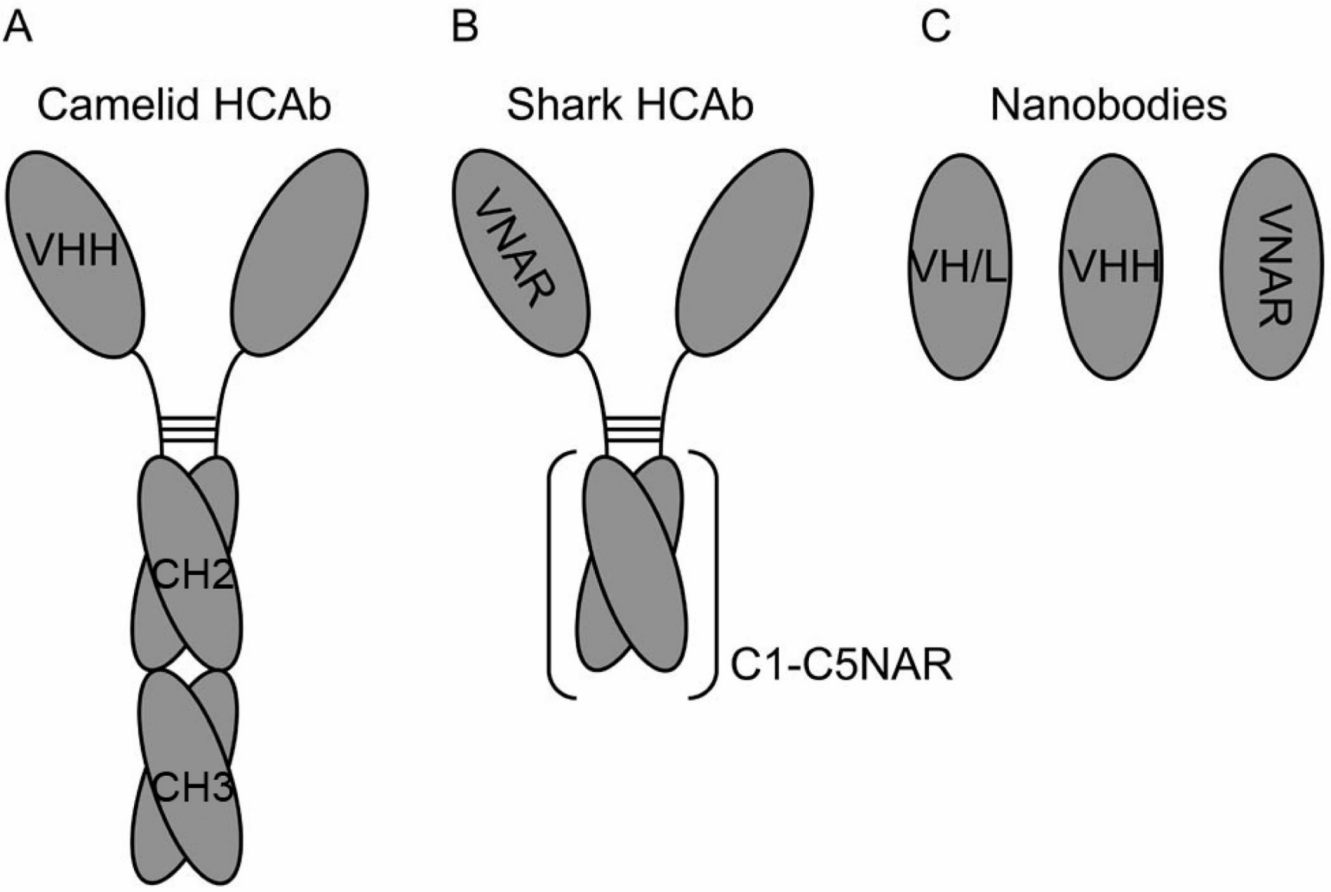
Diagrams depicting the structure of (A) camelid HCAb, (B) shark HCAb, and (C) nanobodies made of VH or VL domains (VH/L), camelid VH domains (VHH) and shark VH domains (VNAR).

Interest was regained after finding that in camelids (alpacas, camels, dromedaries and llamas), and in sharks, part of the their humoral immune response includes Ab molecules that inherently lack light chains [44,45,48]. These heavy-chain Abs (HCAbs) consist of a homodimer of a heavy chain-like polypeptide with either 3 (camelids) or 5 (sharks) Ig domains that compose a single Fc and two identical Fab portions (Figures 4A–B) [49]. The HCAb include just one VH domain per Fab responsible of Ag binding (Figures 4A and B). The camelid VH domains (VHH) and the shark VH domains V-NAR (novel antigen receptor) from the respective HCAbs can be used to generate highly stable nanobodies of minimal size (Figure 4C) (11 – 15 kDa) [50]. Camelid VHH Ig domains are conformationally stable in the absence of a complementary VL [51]. When looking at the sequences found in VHH and V-NAR domains, there are more hydrophilic residues than in VH domains that interface with VL domains [52–54]. Moreover, in some VHH domains it has been shown that hydrophobic residues remaining in the sequences analogous to what would be the VH/VL interface interact with hydrophobic residues in the CDR3 loop [55]. Related with these observations in VHH domains, the human VH domain from the Ab HEL4 is conformationally stable in the absence of its pairing VL domain [56]. Although the interface of HEL4 VH does not present the hydrophilic residues found in camelids, the crystal structure of the single domain reveals that the CDR1 loop interacts with some hydrophobic residues in the VH/VL interface. In line with this emerging concept of reducing hydrophobicity in the Ig domain to gain stability when developing nanobodies, some studies have shown that by randomizing mutations in residues located at the VH/VL interface it was possible to generate mutants of higher stability than the related wild type VH single domain when introducing charged residues [54].

Moving away from the hydrophobic VH/VL interface, a source of instability for the Ig folding in nanobodies is the CDR loops [19,51]. These sequences tend to include hydrophobic residues that contribute to the interaction with the specific epitope. In absence of the epitope, the flexible structure of the CDRs may either stabilize or compromise the folding of an isolated Ig domain. This fact is illustrated by different studies showing that CDRs grafted into Ig domains may either increase or decrease the stability of the resulting hybrid Ig domain [57,58]. On the other hand, VHH and V-NAR domains display high affinity binding to their epitopes, which appears to be supported by a longer CDR3 loop when compared with VH domains. Interestingly, due to their small size and long fingerlike CDR sequences, VHH and V-NAR domains can reach cryptic epitopes deep in cavities (like catalytic sites in enzymes) or large structures that normally escape immunosurveillance by bulkier Ab molecules [49,59].

It seems as if natural evolution of VHH and V-NAR Ig domains is delivering structural solutions for the main limitations that nanobodies present as pharmaceutical drugs (Ig folding stability and Ag affinity). Strong efforts have been directed to either develop nanobodies based on these domains or to modify regular VH domains in an analogous fashion. Libraries of camelid nanobodies have been screened (by either phage, ribosomal or yeast surface displays) in search of high affinity antigenic specificities against multiple targets such enzymes, haptens, viruses, toxins and venoms, cell surface tumor markers, and tissue markers for immune-imaging [60]. Regarding limitations due to their immunogenicity when injected in different species, it has been shown that repeated injection of VHH nanobodies in mice does not stimulate an immune response against them [61,62]. Additionally, immunogenicity of camelid nanobodies has been reduced by sequence humanization methods. The goal in this kind of strategy is to develop a universal humanized nanobody scaffold that upon CDR engraftment from other Abs acquires the related antigen specificity and affinity without loosing its stability [58]. On the other hand, the structure of the CDRs from camelid sdAb suggest the possibility to develop recombinant human VH nanobodies of high affinity by introducing extended CDR1 and CDR3 loops as seen in VHH and V-NAR domains [51]. Another approach to develop new nanobody-based drugs is to find key residues in camelids and sharks responsible of the high solubility and low aggregation of VHH and V-NAR domains. Some studies suggest that camelization of mouse and human VH domains by replacing hydrophobic residues from the complementary surfaces and the structural portions of the CDR loops with negatively charged amino acids might increase conformational stability [63,64].

## 6. ENGINEERING RESISTANCE TO AGGREGATION IN ANTIBODY FRAGMENTS

Once unfolding has taken place and the Ig structure is denatured, the tendency to aggregate depends on the solubility of the denatured Ig domains [14]. When unfolded Ig domains have high solubility, they tend to possess a slow aggregation kinetic, and they are able to refold back to their native conformation when the environmental stress is removed (Figure 2). Maximizing Ig folding stability and solubility is not only important when considering the engineering of single domain Ab fragments like nanobodies, but is also relevant for multi-domain fragments; unfolding of just one domain can drive the aggregation of the whole fragment, while stability of each domain indirectly contributes to the solubility of the whole Ab fragment.

In order to prevent Ig aggregation, significant efforts have been directed to identifying structural determinants in different Ig domains that imprint high solubility in the non-native state. In principle, this knowledge could aid in engineering “super-stable” Ig domains that would remain soluble when exposed to a changing environment and tend to spontaneously refold back into native conformation. Comparison of the sequences of human VH and camelid VHH domains has allowed the identification of residues that in camelids are responsible for the low aggregation (high solubility) of unfolded VHH domains. The so-called VHH tetrad consists of 4 residues found to be key for high solubility in camelid VHH [65]. These amino acids represent substitutions of hydrophobic residues in the human VH domains for either charged or less hydrophobic moieties in the camelid VHH domains. Three of these camelid substitutions occur at the site on VHH where, in the human VH sequence, there would be an interface with VL. The fourth substitution is in the long VHH CDR3 loop. First attempts to introduce the VHH tetrad in human VH only modestly increased the solubility of such domains when unfolded [58]. Better results have been obtained when attempting to humanize camelid VHH domains; high solubility of the unfolded Ig domain is retained when 75% of the sequences remain camelid [14]. When evaluating the role of the CDR sequences in the solubility of human VH domains, one study showed that replacement of one single hydrophobic residue near the CDR1 loop with a charged amino acid imparted solubility to the unfolded VH domain [51]. Solubility was also acquired when mutating three residues in the CDR1 to charged amino acids. These findings are in line with a previous study focused on the high solubility of unfolded VHH domains when compared with human VH domains [66]. In this previous study it was found that the camelid domains displayed two charged residues in the CDR3 loop in positions occupied by hydrophobic residues in the human VH domains. Although a general rule that substitutions of hydrophobic with hydrophilic residues in the CDR loops increases V domain solubility seems to emerge here, it has also been shown that not all substitutions of hydrophobic residues can increase the solubility of Ig domains [51]. Moreover, substitutions of residues along the CDR loops are expected to alter the affinity for binding the specific antigenic epitope. Thus, when considering these kind of alterations to engineer high solubility in Ab fragments, additional efforts should be aimed at preserving epitope binding affinity.

Under different kinds of environmental stress, like low temperature or high protein concentration, Abs and their derived fragments may aggregate [14]. Ig domains of poor solubility under their native conformation may establish homotypic interactions when destabilized by exposure to low temperatures, or when highly concentrated for therapeutic applications (Figure 2). The solubility of native Ig domains is quite variable and it is poorly understood. Even at low protein concentrations, Ab fragments may display some instability that causes formation of aggregates. We have recently described the case of four unrelated anti-TCR/CD3 Fab fragments that dimerize at low protein concentrations (0.2 mg/ml) while preserving their Ag binding specificity [67]. Fab dimerization occurred for all four fragments studied under conditions of storage in standard phosphate-buffered saline (PBS). Other studies point toward a possible role for the CDR loops and their hydrophobic residues in reducing the solubility of native Ig domains. One strategy to increase native Ig solubility without mutating the hydrophobic residues at the CDRs was tested by Feng and colleagues. These authors applied the general concept of increasing Ab solubility by adding oligosaccharides [68], and introduced one glycosylation site inside the CDR2 to compensate for some hydrophobic residues in the CDR3 of a specific Ab. The glycosylation site was strategically located to not interfere with the CDR portions interacting with the Ag. In this way the solubility of the glycosylated Ab was increased when compared with the wild type Ab, without perturbing the affinity for the specific Ag [69]. In other cases, the environment causes instability precisely at the hydrophobic interface of complementary Ig domains like VH and VL. In this situation intermolecular Ig swapping may be promoted in order to regain stability [37]. As mentioned above, Ig swapping is the mechanism of scFv oligomerization when short linkers are used to impede the intra-molecular association of complementary VH/VL domains [37,38]. However it has been shown that environmental conditions like antigen presence, pH, and ionic strength can drive Ig domain swapping in scFv fragments independently of the length of their linker [26,37]. Strategies to inhibit undesired Ig swapping in monomeric scFv fragments are based on increasing the stability of the VH/VL interface by either engineering disulfide bonds, as in the sc-dsFv fragments [26], or introducing charged mutations [70]. Ig swapping in Fab fragments and intact antibody molecules is considered rare [71], and it has not been observed among hundreds of Fab structures reported in the Protein Data Base [35]. Yet, there is at least one report of a functional form of antibody that consists of a dimer of swapped of VH domains. The human mAb 2G12, which binds a cluster of the disaccharide Manα1–2Man and the oligosaccharide Man9GlcNAc2, recognizes its sugar epitopes in the HIV protein gp120 by an extended binding site that includes the swapping of the VH domains between two antibody molecules [72].

## 7. USE OF OSMOLYTES TO PREVENT Ig UNFOLDING AND AGGREGATION

Natural osmolytes are small organic molecules including certain amino acids and derivates (*i.e*., glycine, alanine, lysine, proline, taurine), methylamines (*i.e*., betaine, sarcosine trimethylamine N-oxide (TMAO)), polyalcohols and sugars (*i.e*., glycerol), and urea. Osmolytes accumulate inside the cell to counteract osmotic stress [24,73–75]. When cells get exposed to dramatic changes in temperature, high concentrations of salts, or desiccation, they augment their intracellular concentration of osmolytes to increase their osmotic pressure and avoid loss of water. Osmolytes present high solubility in water, and they can reach the range of molar concentrations in the cytoplasm [76,77]. Osmolytes are described as compatible solutes since they do not covalently modify other molecules, and therefore are not thought to alter physiologic cellular processes [78]. Osmolytes display a second role related with osmotic stress, in that they increase the solubility of intracellular proteins [79]. Inside the cell, proteins must remain folded to maintain their functionality. Throughout evolution, different organisms from bacteria to mammals have selected osmolytes that impact the folding equilibrium of proteins favoring their functional native conformation, and preventing loss of function under environmental stress. These osmolytes are classified as protecting (*i.e*., arginine, proline, TMAO) because they increase protein solubility and maintenance of protein native state, inhibiting protein aggregation (Figure 2). Alternatively, denaturing osmolytes (*i.e*., urea) have an opposite effect, solubilizing proteins to a denatured state. The protective vs. denaturing character of osmolytes relies on their solvophobic/solvophylic interactions with the peptide backbone of proteins [24,80–82]. In the case of protecting osmolytes, there is a solvophobic effect consisting of repulsion forces between the peptide backbone of the protein and the osmolyte in solution that favors intra-molecular hydrogen bonds along the polypeptide, which in turn stabilizes the secondary structure and native conformation of the protein. The solvophobic effect drives polypeptides to exclude osmolyte molecules from the protein surface by stabilizing a compact, folded conformation that minimizes their solute-exposed surface area [77,79]. In the case of denaturing osmolytes, direct interactions between the osmolyte and the peptide backbone interfere with protein intra-molecular interactions, destabilizing their secondary structure and resulting in unfolding.

Among the natural protecting osmolytes, there are examples of osmolytes that preserve protein folding under low (arginine, aspartate, glycine, glutamate, histidine, lysine) or high (sucrose, trehalose) temperatures, high concentration of salts (glycerol, proline), exposure to urea (sarcosine, TMAO), and desiccation (trehalose, sucrose) [76]. Interestingly, during manufacturing, storage, reconstitution and administration of drugs composed of Ab fragments, the Ig domains may get exposed to changes in their environmental conditions that may drive their unfolding. As the Ig domain becomes unstable and starts unfolding, aggregation may occur. As discussed above, unfolding and aggregation of the Ig domain is an undesirable event that may cause an irreversible loss of therapeutic effects, together with acquisition of immunogenicity and/or toxicity. As discussed in previous sections, much can be done to optimize the stability of Ig domains by manipulation of the polypeptide sequences of Ab fragments. Yet, the capacity of osmolytes to promote protein folding, packing, and conformation stability provides additional strategies to prevent the aggregation of Ab based proteins during the different stages of their production, distribution, storage, and usage [83–85]. Osmolytes like betaine, glycerol, glycine and proline have been used to optimize the yield of different recombinant proteins and immunotoxins synthetized in *E. coli* as soluble native products [76,86]. In other cases, production of recombinant proteins in E. coli leads to their accumulation as insoluble aggregates in inclusion bodies (IB). Osmolytes like proline have been used to help refold proteins into their native conformation, once solubilizing agents (arginine, guanidine, SDS, urea) have been removed [87]. Osmolytes like glycine, lysine and histidine have been shown to prevent aggregation of recombinant growth factors and mAbs under heat stress [88–90]. Arginine, aspartic acid and histidine stabilize Ab molecules during lyophilization [91], while glutamic acid, glycine and lysine prevent aggregation of lyophilized IL2 and keratinocyte growth factor when they are re-hydrated [92,93]. As described above, protecting osmolytes favor the most compact state in proteins by the repulsion effect against an extended peptide backbone linked to the unfolded state. In vitro and in vivo, proline has been shown to prevent aggregation of two different model proteins prone to aggregation under osmotic stress by destabilizing partially unfolded states and small aggregates [94] (Figure 2). We have described recently the prevention and a partial reversion of dimerization of Fab fragments from four unrelated anti-TCR/CD3 when diluted in a PBS/proline 2 M buffer [67]. Interestingly, dimeric Fabs maintained full Ag recognition when compared with monomer Fabs, indicating a case of Ig association with preservation of functional (native) folding (Figure 2).

## 8. CONCLUDING REMARKS

An increased understanding of the mechanisms by which the Ig domain folds and retains its native/functional conformation will aid continued efforts to generate Ab-based therapeutics and diagnostics. In this review we have discussed the most recent findings regarding these questions that allow the development of different approaches to achieve stable Ig domains compatible with the manufacturing and commercialization of Ab fragment-based biotechnologies. Still better understanding of the Ig folding, regarding its stability and solubility in relation to changing environmental conditions is required to achieve the ultimate goal, obtaining a universal Ig scaffold that can function as a stable building block for different types of Ab fragments.
